# A new insular species of the *Cyrtodactyluspulchellus* group (Reptilia, Gekkonidae) from Tarutao Island, southern Thailand revealed by morphological and genetic evidence

**DOI:** 10.3897/zookeys.1070.73659

**Published:** 2021-10-12

**Authors:** Korkhwan Termprayoon, Attapol Rujirawan, Natee Ampai, Perry L. Wood Jr, Anchalee Aowphol

**Affiliations:** 1 Department of Zoology, Faculty of Science, Kasetsart University, Bangkok, 10900 Thailand Kasetsart University Bangkok Thailand; 2 Department of Biology, Faculty of Science, Srinakharinwirot University, Bangkok, 10110 Thailand Srinakharinwirot University Bangkok Thailand; 3 Department of Biological Sciences and Museum of Natural History, Auburn University, Auburn, AL, USA Auburn University Auburn United States of America; 4 Department of Ecology and Evolutionary Biology, University of Michigan, Ann Arbor, MI 48109-1085, USA University of Michigan Ann Arbor United States of America

**Keywords:** *Cyrtodactylusastrum*, *Cyrtodactylusstellatus* sp. nov., karst, morphology, phylogeny, taxonomy

## Abstract

The bent-toed geckos of the *Cyrtodactyluspulchellus* group are widely distributed along the Thai-Malay Peninsula. Although taxonomic and phylogenetic studies of this species group have been continuously conducted, only some populations from Thailand have been included, resulting in hidden diversity within this group. In this study, we used morphological and molecular data to clarify the taxonomic status and describe a new population from Tarutao Island, Satun Province, southern Thailand. *Cyrtodactylusstellatus***sp. nov.** can be distinguished from its congeners by the combination of the following morphological characters: body size; tuberculation; number of dark body bands, ventral scales, and femoroprecloacal pores in males; presence of precloacal pores in females; and scattered pattern on dorsum. Phylogenetic analyses of the mitochondrial ND2 gene recovered the new species as the sister species to *C.astrum*, with an uncorrected pairwise divergence of 9.78–12.37%. *Cyrtodactylusstellatus***sp. nov.** is currently only known from Tarutao Island, Thailand. The discovery of this species suggests that the diversity within the *C.pulchellus* group remains underestimated and future exploration of unsurveyed areas are needed to further the understanding of this group and its geographic range.

## Introduction

Bent-toed geckos in the genus *Cyrtodactylus* Gray, 1827 are geographically widespread and inhabit lowland (e.g., peat swamps, karst formations, and limestone forests) to mountainous regions (> 1,500 m a.s.l) of South Asia to Melanesia, ranging from India, Myanmar, Thailand, Vietnam, Cambodia, Malaysia, Java, Papua New Guinea to northern Australia (Wood et al. 2012; Nielsen and Oliver 2017; Pauwels et al. 2018; Purkayastha et al. 2020; Riyanto et al. 2020; Grismer et al. 2020a, 2021a, 2021b). This genus is the most diverse group of gekkotans, comprising 314 nominal species (Uetz et al. 2021). During the last two decades, the number of new species described in this genus has significantly increased with the exploration of unsurveyed karst formations (Luu et al. 2016; Nazarov et al. 2018; Davis et al. 2019; Grismer et al. 2018, 2020b). Moreover, genetic data has become a useful tool for taxonomic studies, revealing hidden diversity within the genus (Murdoch et al. 2019; Chomdej et al. 2020; Neang et al. 2020; Riyanto et al. 2020; Kamei and Mahony 2021; Liu and Rao 2021). Recent molecular studies have further supported the monophyly of this genus based on the most complete phylogenetic analysis to date, and have recognized 31 species groups (Grismer et al. 2021b).

One clade of particular interest is the *Cyrtodactyluspulchellus* group. This relatively diverse group is distributed along the Thai-Malay Peninsula and has high morphological and molecular variation. *Cyrtodactyluspulchellus* Gray, 1827 was thought to be a single wide-ranging species across their distributional range, but following an integrative approach many new species have been described (e.g., *C.bintangrendah*Grismer et al., 2012, *C.langkawiensis*Grismer et al., 2012, and *C.sharkari*Grismer et al., 2014). This species group has been recovered as monophyletic and currently contains 16 recognized species, based on multiple phylogenetic studies (Grismer et al. 2012, 2014, 2016; Quah et al. 2019; Wood et al. 2020; Termprayoon et al. 2021). This group is distributed from the south of the Isthmus of Kra, southern Thailand to southern Peninsular Malaysia and some of its offshore islands (Grismer and Ahmad 2008; Sumontha et al. 2012; Grismer et al. 2012, 2014, 2016; Quah et al. 2019; Wood et al. 2020; Termprayoon et al. 2021). During field surveys, specimens of the *C.pulchellus* group were collected from Tarutao Island, Satun Province, southern Thailand. Initially, these specimens were recognized as an insular population of *C.astrum*Grismer et al., 2012 due to their superficial resemblance in coloration pattern and dorsal tuberculation. A re-examination of these specimens showed morphological differences from its other congeners and mitochondrial DNA sequence data revealed corroborative evidence that the new population of *Cyrtodactylus* from Tarutao Island represents a distinct monophyletic lineage and is the sister species to *C.astrum* from the adjacent mainland. Based on integrative analyses, we considered this new *Cyrtodactylus* population from Tarutao Island as distinct and described it as a new species below.

## Materials and methods

### Sampling

**Figure 1.**
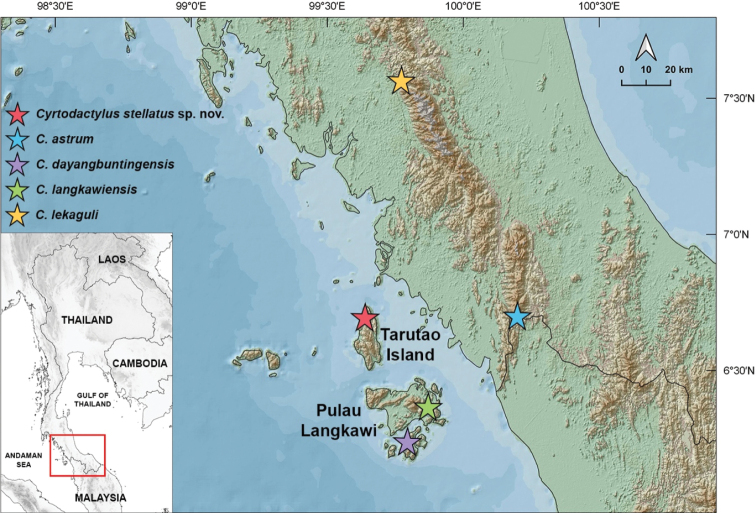
Map showing the type locality of *Cyrtodactylusstellatus* sp. nov. from Tarutao Island, Mueang Satun District, Satun Province, Thailand and the type localities of closely related species, *C.astrum, C.dayangbuntingensis, C.langkawiensis*, and *C.lekaguli*.

Field surveys were conducted on Tarutao Island, Mueang Satun District, Satun Province, southern Thailand from November 2017 to November 2019 (Fig. 1). Specimens of the *C.pulchellus* group were collected from karst forest at night (1900–2200 h). Ecological data (air temperature and relative humidity) were recorded using a Kestrel 4000 Weather Meter, and habitat use of each specimen was noted. Geographical coordinates and elevation were recorded using a Garmin GPSMAP 64s. For molecular studies, liver tissue was taken from each euthanized specimen, individually preserved in 95% ethyl alcohol, and stored at -20 °C. Specimens were initially fixed in 10% formalin and later transferred into 70% ethyl alcohol for permanent storage. Voucher specimens were deposited in the herpetological collections of the Zoological Museum, Kasetsart University, Thailand (ZMKU). Additional preserved specimens were examined in the holdings of the Thailand Natural History Museum (THNHM), Thailand, and the La Sierra University Herpetological Collection (LSUHC), La Sierra University, Riverside, California, USA.

### DNA extraction and PCR amplification

Total genomic DNA was extracted from ethanol-preserved liver tissue of five *Cyrtodactylus* specimens from Tarutao Island (Table 1) using a NucleoSpin Tissue Kit (Macherey-Nagel GmbH & Co. KG, Germany). A fragment of mitochondrial NADH dehydrogenase subunit 2 (ND2) gene and its flanking tRNAs was amplified using a double-stand Polymerase Chain Reaction (PCR) under the following conditions: initial denaturation at 94 °C for 4 min, followed by 35 cycles of denaturation at 94 °C for 30 sec, annealing at 48–52 °C for 30 sec, extension at 72 °C for 1 min 30 sec, and final extension at 72 °C for 7 min using the primers Metf6 (5' AAGCTTTCGGGCCCATACC 3'; Macey et al. 1997), and COIH (5' AGRGTGCCAATGTCTTTGTGRTT 3'; Macey et al. 1997). PCR products were purified using NucleoSpin Gel and PCR Clean-Up kit (Macherey-Nagel GmbH & Co. KG, Germany). Purified products were sequenced for both strands using the same amplifying primers on an ABI 3730XL DNA Sequencer (Applied Biosystems, CA, USA). Sequences were visually checked and aligned in Geneious R11 (Biomatters, Ltd, Auckland, New Zealand). The protein-coding region of ND2 was translated to amino acids and checked to confirm the lack of premature stop codons. All sequences were deposited in GenBank under the accession numbers OK094494–OK094503 (Table 1).

**Table 1.** Samples used in the molecular analyses, including their GenBank accession number (ND2), voucher number and locality. WM = West Malaysia; TH = Thailand.

Species	Locality	Museum No.	GenBank Accession No.	Reference
**Outgroup**				
*Agamurapersica*	Pakistan, Baluchistan Province, Makran District, Gwadar division	FMNH 247474	JX440515	Wood et al. (2012)
*Hemidactylusfrenatus*	Unknow	NC 00155	JX519468	Grismer et al. (2012)
*Tropiocolotessteudneri*	captive	JB 28	JX440520	Wood et al. (2012)
*C.elok*	WM, Pahang, Fraser’s Hill, The Gap	LSUHC 6471	JQ889180	Wood et al. (2012)
*C.hontreensis*	Vietnam, Kien Giang Province, Kien Hai District, Hon Tre Island	LSUHC 8583	JX440539	Wood et al. (2012)
*C.intermedius*	TH, Chantaburi Province, Khao Khitchakut District	LSUHC 9513	JX519469	Grismer et al. (2012)
	TH, Chantaburi Province, Khao Khitchakut District	LSUHC 9514	JX519470	Grismer et al. (2012)
*C.interdigitalis*	Lao, Khammouan Province, Nakai District	FMNH 255454	JQ889181	Wood et al. (2012)
*Cyrtodactylus* sp.	TH, Loei, Phu Rua	FMNH 265806	JX519471	Grismer et al. (2012)
**Ingroup**				
*C.astrum*	WM, Perlis, Gua Kelam	LSUHC 8806	JX519481	Grismer et al. (2012)
	WM, Perlis, Gua Kelam	LSUHC 8807	JX519478	Grismer et al. (2012)
	WM, Perlis, Gua Kelam	LSUHC 8808	JX519479	Grismer et al. (2012)
	WM, Perlis, Gua Kelam	LSUHC 8809	JX519480	Grismer et al. (2012)
	WM, Perlis, Kuala Perlis	LSUHC 8815	JX519482	Grismer et al. (2012)
	WM, Perlis, Kuala Perlis	LSUHC 8816 (paratype)	JX519483	Grismer et al. (2012)
	WM, Perlis, Perlis State Park	LSUHC 9215	JX519473	Grismer et al. (2012)
	WM, Perlis, Perlis State Park, Gua Wang Burma	LSUHC 9962	JX519475	Grismer et al. (2012)
	WM, Perlis, Perlis State Park, Gua Wang Burma	LSUHC 9986	JX519476	Grismer et al. (2012)
	WM, Perlis, Perlis State Park, Gua Wang Burma	LSUHC 9987	JX519477	Grismer et al. (2012)
	WM, Perlis, Wang Kelian	LSUHC 10023	JX519474	Grismer et al. (2012)
	WM, Perlis, Wang Kelian	LSUHC 10024	JX519472	Grismer et al. (2012)
*C.australotitiwangsaensis*	WM, Pahang, Fraser’s Hill	LSUHC 8086	JX519486	Grismer et al. (2012)
	WM, Pahang, Fraser’s Hill	LSUHC 8087	JX519485	Grismer et al. (2012)
	WM, Pahang, Genting Highlands	LSUHC 6637 (holotype)	JX519484	Grismer et al. (2012)
*C.bintangrendah*	WM, Kedah, Bukit Mertajam	LSUHC 10331 (paratype)	MN125076	Quah et al. (2019)
	WM, Kedah, Bukit Mertajam	LSUHC 10519	MN125077	Quah et al. (2019)
	WM, Kedah, Bukit Mertajam	LSUHC 10520 (paratype)	MN125078	Quah et al. (2019)
	WM, Kedah, Bukit Palang	LSUHC 9984	JX519487	Grismer et al. (2012)
*C.bintangtinggi*	WM, Perak, Bukit Larut	LSUHC 8862	JX519493	Grismer et al. (2012)
	WM, Perak, Bukit Larut	LSUHC 9006 (paratype)	JX519494	Grismer et al. (2012)
*C.dayangbuntingensis*	WM, Kedah, Dayang Bunting Island	LSUHC 14353	MN125090	Quah et al. (2019)
	WM, Kedah, Dayang Bunting Island	LSUHC 14354	MN125091	Quah et al. (2019)
	WM, Kedah, Dayang Bunting Island	LSUHC 14355	MN125092	Quah et al. (2019)
*C.evanquahi*	WM, Kedah, Gunung Baling	BYU 53435 (holotype)	MN586889	Wood et al. (2020)
	WM, Kedah, Gunung Baling	BYU 53436 (paratype)	MN586890	Wood et al. (2020)
	WM, Kedah, Gunung Baling	BYU 53437 (paratype)	MN586891	Wood et al. (2020)
*C.hidupselamanya*	WM, Kelantan, Felda Chiku 7	LSUHC 12161 (paratype)	KX011415	Grismer et al. (2016)
	WM, Kelantan, Felda Chiku 7	LSUHC 12162 (paratype)	KX011416	Grismer et al. (2016)
	WM, Kelantan, Felda Chiku 7	LSUHC 12163 (holotype)	KX011417	Grismer et al. (2016)
	WM, Kelantan, Felda Chiku 7	LSUHC 12173 (paratype)	KX011420	Grismer et al. (2016)
*C.jelawangensis*	WM, Gunung Stong, Kelantan	LSUHC 11060 (paratype)	KJ659850	Grismer et al. (2014)
	WM, Gunung Stong, Kelantan	LSUHC 11062 (holotype)	KJ659852	Grismer et al. (2014)
	WM, Kelantan, Gunung Stong	LSUHC 11061 (paratype)	KJ659851	Grismer et al. (2014)
*C.langkawiensis*	WM, Kedah, Pulau Langkawi, Wat Wanaram	LSUHC 9120	JX519502	Grismer et al. (2012)
	WM, Kedah, Pulau Langkawi, Wat Wanaram	LSUHC 9122	JX519501	Grismer et al. (2012)
	WM, Kedah, Pulau Langkawi, Wat Wanaram	LSUHC 9123	JX519500	Grismer et al. (2012)
	WM, Kedah, Pulau Langkawi, Wat Wanaram	LSUHC 9124 (paratype)	JX519499	Grismer et al. (2012)
	WM, Kedah, Pulau Langkawi, Wat Wanaram	LSUHC 9125	JX519496	Grismer et al. (2012)
	WM, Kedah, Pulau Langkawi, Wat Wanaram	LSUHC 9434	JX519498	Grismer et al. (2012)
	WM, Kedah, Pulau Langkawi, Wat Wanaram	LSUHC 9435	JX519495	Grismer et al. (2012)
	WM, Kedah, Pulau Langkawi, Wat Wanaram	LSUHC 9437	JX519497	Grismer et al. (2012)
	WM, Kedah, Pulau Langkawi, Wat Wanaram	LSUHC 14347	MN125093	Quah et al. (2019)
	WM, Kedah, Pulau Langkawi, Wat Wanaram	LSUHC 14348	MN125094	Quah et al. (2019)
*C.lekaguli*	TH, Phang-nga Province, Mueang Phang-nga District	ZMKU R 00720	KX011425	Grismer et al. (2016)
	TH, Phang-nga Province, Mueang Phang-nga District	ZMKU R 00721	KX011426	Grismer et al. (2016)
	TH, Phang-nga Province, Mueang Phang-nga District	ZMKU R 00722	KX011427	Grismer et al. (2016)
	TH, Phang-nga Province, Mueang Phang-nga District	ZMKU R 00723	KX011428	Grismer et al. (2016)
	TH, Trang Province, Na Yong District	ZMKU R 00918	OK094494	This study
	TH, Trang Province, Na Yong District	ZMKU R 00919	OK094495	This study
	TH, Trang Province, Na Yong District	ZMKU R 00920	OK094496	This study
	TH, Trang Province, Na Yong District	ZMKU R 00921	OK094497	This study
	TH, Trang Province, Na Yong District	ZMKU R 00922	OK094498	This study
*C.lenggongensis*	WM, Perak, Lenggong Valley	LSUHC 9974 (holotype)	JX519490	Grismer et al. (2012)
	WM, Perak, Lenggong Valley	LSUHC 9975 (paratype)	JX519488	Grismer et al. (2012)
	WM, Perak, Lenggong Valley	LSUHC 9976 (paratype)	JX519489	Grismer et al. (2012)
	WM, Perak, Lenggong Valley	LSUHC 9977 (paratype)	JX519491	Grismer et al. (2012)
*C.macrotuberculatus*	TH, Phuket Province, Kathu District, Kathu Waterfall	ZMKU R 00890	MW809301	Termprayoon et al. (2021)
	TH, Phuket Province, Kathu District, Kathu Waterfall	ZMKU R 00891	MW809302	Termprayoon et al. (2021)
	TH, Phuket Province, Thalang District, Thep Krasatti	ZMKU R 00894	MW809305	Termprayoon et al. (2021)
	TH, Phuket Province, Thalang District, Thep Krasatti	ZMKU R 00895	MW809306	Termprayoon et al. (2021)
	TH, Phuket Province, Thalang District, Thep Krasatti	ZMKU R 00896	MW809307	Termprayoon et al. (2021)
	TH, Satun Province, Mueang Satun District, Adang Island	ZMKU R 00875	MW809295	Termprayoon et al. (2021)
	TH, Satun Province, Mueang Satun District, Rawi Island	ZMKU R 00883	MW809299	Termprayoon et al. (2021)
	TH, Satun Province, Mueang Satun District, Rawi Island	ZMKU R 00887	MW809300	Termprayoon et al. (2021)
	TH, Songkhla Province, Hat Yai District, Thung Tam Sao	ZMKU R 00876	MW809296	Termprayoon et al. (2021)
	TH, Songkhla Province, Hat Yai District, Thung Tam Sao	ZMKU R 00877	MW809297	Termprayoon et al. (2021)
	TH, Songkhla Province, Hat Yai District, Thung Tam Sao	ZMKU R 00878	MW809298	Termprayoon et al. (2021)
	WM, Kedah, Hutan Lipur Sungai Tupah	LSUHC 9671	JX519510	Grismer et al. (2012)
	WM, Kedah, Hutan Lipur Sungai Tupah	LSUHC 9672	JX519511	Grismer et al. (2012)
	WM, Kedah, Hutan Lipur Sungai Tupah	LSUHC 9693	JX519517	Grismer et al. (2012)
	WM, Kedah, Pulau Langkawi, Gunung Machinchang	LSUHC 9448	JX519507	Grismer et al. (2012)
*C.macrotuberculatus*	WM, Kedah, Pulau Langkawi, Gunung Raya	LSUHC 9428	JX519506	Grismer et al. (2012)
	WM, Kedah, Pulau Langkawi, Lubuk Sembilang	LSUHC 6829	JX519505	Grismer et al. (2012)
	WM, Perlis, Bukit Chabang	LSUHC 10037	JX519519	Grismer et al. (2012)
	WM, Perlis, Bukit Chabang	LSUHC 10038	JX519518	Grismer et al. (2012)
*C.pulchellus*	WM, Penang, Pulau Pinang, Empangan Air Itam	LSUHC 6668	JX519523	Grismer et al. (2012)
	WM, Penang, Pulau Pinang, Moongate Trail	LSUHC 6727	JX519526	Grismer et al. (2012)
	WM, Penang, Pulau Pinang, Moongate Trail	LSUHC 6728	JX519525	Grismer et al. (2012)
	WM, Penang, Pulau Pinang, Moongate Trail	LSUHC 6729	JX519528	Grismer et al. (2012)
*C.sharkari*	WM, Pahang, Merapoh, Gua Gunting	LSUHC 11022 (holotype)	KJ659853	Grismer et al. (2014)
*Cyrtodactylusstellatus* sp. nov.	TH, Satun Province, Mueang Satun District, Tarutao Island	ZMKU R 00903 (paratype)	OK094499	This study
	TH, Satun Province, Mueang Satun District, Tarutao Island	ZMKU R 00905 (holotype)	OK094500	This study
	TH, Satun Province, Mueang Satun District, Tarutao Island	ZMKU R 00906 (paratype)	OK094501	This study
	TH, Satun Province, Mueang Satun District, Tarutao Island	ZMKU R 00907 (paratype)	OK094502	This study
	TH, Satun Province, Mueang Satun District, Tarutao Island	ZMKU R 00908 (paratype)	OK094503	This study
*C.timur*	WM, Gunung Tebu, Terengganu	LSUHC 10886	KJ659854	Grismer et al. (2014)
	WM, Gunung Tebu, Terengganu	LSUHC 11183 (paratype)	KJ659855	Grismer et al. (2014)
	WM, Gunung Tebu, Terengganu	LSUHC 11184 (paratype)	KJ659856	Grismer et al. (2014)
	WM, Gunung Tebu, Terengganu	LSUHC 11185 (paratype)	KJ659857	Grismer et al. (2014)
*C.trilatofasciatus*	WM, Pahang, Cameron Highlands	LSUHC 10064	JX519529	Grismer et al. (2012)
	WM, Pahang, Cameron Highlands	LSUHC 10065	JX519530	Grismer et al. (2012)
	WM, Pahang, Cameron Highlands	LSUHC 10066	JX519531	Grismer et al. (2012)

### Phylogenetic analyses

Phylogenetic trees were reconstructed using two different methods, Maximum Likelihood (ML) and Bayesian Inference (BI). The best substitution model for each partition was determined using the Bayesian Information Criterion (BIC) under the greedy search algorithm as implemented in PartitionFinder2 on XSEDE (Lanfear et al. 2016). The selected models for ML and BI were TIM+G for 1^st^ and 2^nd^ codon positions of ND2, TVM+I+G for 3^rd^ codon position of ND2 and TRN+I+G for tRNAs. The ML analysis was performed in IQ-TREE web server v1.6.12 (Trifinopoulos et al. 2016) with 1,000 bootstrap replicates using ultrafast bootstrap approximation (Minh et al. 2013). The BI analysis was performed in MrBayes 3.2.6 on XSEDE (Ronquist et al. 2012) using the CIPRES Science Gateway v3.3 (Miller et al. 2010). Two simultaneous runs were performed with four chains per run, three hot and one cold under the default settings. The analysis was run for 10,000,000 generations and sampled every 1,000 generations from the Markov chain Monte Carlo (MCMC), with the first 25% of each run discarded as burn-in. Stationarity and the effective sample sizes (ESS) for all parameters were assessed in Tracer v1.7.1. (Rambaut et al. 2018). Nodes with ultrafast bootstrap support (UFB) of ≥ 95 and Bayesian posterior probabilities (BPP) of ≥ 0.95 were considered to be strongly supported (Huelsenbeck and Ronquist 2001; Wilcox et al. 2002; Minh et al. 2013). Intraspecific and interspecific uncorrected pairwise genetic divergences (*p*-distance) were calculated in MEGA X 10.0.5 using the pairwise deletion option for the treatment of gaps and missing data in the dataset (Kumar et al. 2018).

### Morphology

The morphological characters and their definition used in this study were modified from previous studies of the *C.pulchellus* group (Grismer and Ahmad 2008; Grismer et al. 2012, 2014, 2016; Quah et al. 2019; Wood et al. 2020), and abbreviations are derived from Grismer et al. (2018, 2020c). All mensural characters were taken with digital calipers to the nearest 0.01 mm on the left side, while scale counts were made on both sides when possible. Scalation and external morphology were evaluated under a Nikon SMZ745 dissecting microscope. Measurement and meristic characters are shown in Table 2, and external morphological characters evaluated are described below.

**Table 2.** Measurement and meristic characters used in this study, with abbreviations and explanations.

Abbreviations	Characters
**Measurement**	
**SVL**	Snout–vent length, taken from the tip of snout to the vent
**TW**	Tail width, taken at the base of the tail immediately posterior to the postcloacal swelling
**TL**	Tail length, taken from vent to the tip of the tail, original or regenerated
**FL**	Forearm length, taken from the posterior margin of the elbow while flexed 90º to the inflection of the flexed wrist
**TBL**	Tibia length, taken from the posterior surface of the knee while flexed 90º to the base of the heel
**AG**	Axilla to groin length, taken from the posterior margin of the forelimb at its insertion point on the body to the anterior margin of the hind limb at its insertion point on the body
**HL**	Head length, the distance from the posterior margin of the retroarticular process of the lower jaw to the tip of the snout
**HW**	Head width, measured at the angle of the jaws
**HD**	Head depth, the maximum height of head from the occiput to the throat
**ED**	Eye diameter, the greatest horizontal diameter of the eyeball
**EE**	Eye to ear distance, measured from the anterior edge of the ear opening to the posterior edge of the eyeball
**ES**	Eye to snout distance, measured from anterior most margin of the eyeball to the tip of snout
**EN**	Eye to nostril distance, measured from the anterior margin of the eyeball to the posterior margin of the external nares
**IO**	Inter orbital distance, measured between the anterior edges of the orbit
**EL**	Ear length, the greatest vertical distance of the ear opening
**IN**	Internarial distance, measured between the nares across the rostrum
**Meristic**	
**SL**	Supralabial scales, counted from the largest scale immediately posterior to the dorsal inflection of the posterior portion of the upper jaw to the rostral scale
**IL**	Infralabial scales, counted from the largest scale immediately posterior to the dorsal inflection of the posterior portion of the upper jaw to the mental scale
**PVT**	The number of paravertebral tubercles between limb insertions, counted in a straight line immediately left or right of the vertebral column
**LRT**	The number of longitudinal rows of body tubercles, counted transversely across the center of the dorsum from one ventrolateral fold to the other
**VS**	The number of longitudinal rows of ventral scales, counted transversely across the center of the abdomen from one ventrolateral fold to the other
**4TL**	The number of subdigital lamellae beneath the fourth toe, counted from the base of the first phalanx to the claw
**FPP**	The total number of precloacal and femoral pores in male (i.e., the sum of the number of femoral and precloacal scales bearing pores combined as a single meristic referred to as the femoroprecloacal pores)
**PP**	The number of precloacal pores in female
**BB**	The number of dark body bands between limb insertions
**DCB**	The number of dark caudal bands on the original tail

External morphological characters examined in the *C.pulchellus* group were the degree of body tuberculation, weak tuberculation referring to dorsal body tubercles that are low and rounded whereas prominent tuberculation refer to tubercles that are raise and keeled; the presence or absence of tubercles on the dorsal and ventral surface of the forearms; the presence or absence of tubercles in the gular region, throat, and ventrolateral body folds; the width of the dark body bands relative to the width of the interspace between the bands; the presence or absence of dark pigmentation infused in the white caudal bands of adults; the presence or absence of a precloacal depression or groove; the presence or absence of scattered white/yellow tubercles on the dorsum; and the presence or absence of white tail tip on the posterior portion of the original tail in hatchlings and juveniles. Color pattern characteristics were taken from digital images of live specimens in both sexes and of all possible age classes prior to preservation.

### Statistical analyses

All analyses were performed using the base statistical software in R v3.6.1 (R Core Team 2019). To eliminate bias of sexual dimorphism, adult males and females were analyzed separately. Morphological analyses were run on 15 mensural characters. Tail length (TL) was not included due to their different condition (e.g., original, regenerated, and broken). All measurements of each species were size-adjusted in order to remove potential effects of allometry using the following allometric equation: X_adj_ = log[X ± β(SVL ± SVL_mean_)], where X_adj_ = adjusted value; X = measured value; β = unstandardized regression coefficient for each OTU; SVL = measured snout–vent length; SVL_mean_ = overall average SVL of each OTU (Thorpe 1975, 1983; Turan 1999; Lleonart et al. 2000)—implemented through the R package GroupStruct (Chan and Grismer 2021). Morphological measurements of *C.astrum, C.dayangbuntingensis*Quah et al., 2019, *C.langkawiensis* and *C.lekaguli*Grismer et al., 2012 were obtained from their original descriptions (Grismer et al. 2012; Quah et al. 2019). Additional preserved specimens of *C.astrum* (from Malaysia) and *C.lekaguli* (topotypes) were examined and included in the analyses (Appendix I). Morphometric adjustments were conducted separately on each species and then concatenated into a single data frame to ensure there was no interspecific conflation of variation (Reist 1985; McCoy et al. 2006). Specimens were assigned into five groups (= species) based on phylogenetic analyses which are *Cyrtodactylus* Tarutao Island samples (*N* = 5 males, 5 females), *C.astrum* (*N* = 5 males, 3 females), *C.dayangbuntingensis* (*N* = 2 males), *C.langkawiensis* (*N* = 2 males, 4 females), and *C.lekaguli* (*N* = 7 males, 9 females).

Principal components analysis (PCA) was performed on size-adjusted data for each sex using FactoMineR package (Lê et al. 2008) and were visualized with the R package ggplot2 (Wickham 2016). For univariate analysis, Shapiro-Wilk test was used to evaluate data to meet normality assumptions (*p* ≥ 0.05) and Levene’s test for testing for equality of variance (*p* ≥ 0.05). Morphological differences were compared using Analysis of variance (ANOVA) or Kruskal-Wallis test. ANOVA was conducted on normally distributed data with homogeneous variances and were subjected to Tukey HSD post hoc tests (Tukey’s test) to determine which characters had statistically different mean values for which pairs of species if ANOVA had a *p*-value of less than 0.05. Kruskal-Wallis test was performed on non-normally distributed data and followed by a post hoc Dunn’s multiple comparison (Dunn’s test). Due to limited sample sizes, *C.dayangbuntingensis* (*N* = 2 males) and males of *C.langkawiensis* (*N* = 2 males) were excluded from the univariate analysis.

## Results

### Phylogenetic relationships

The aligned matrix contained 1,429 mtDNA characters from 93 individuals of the *C.pulchellus* group and nine individuals of outgroup species (Table 1). The standard deviation of split frequencies among the two simultaneous BI runs was 0.002676, and the ESS values of all parameters were greater than or equal to 2,494.4. The maximum likelihood value of the best ML tree was lnL = -15,115.412.

**Figure 2.**
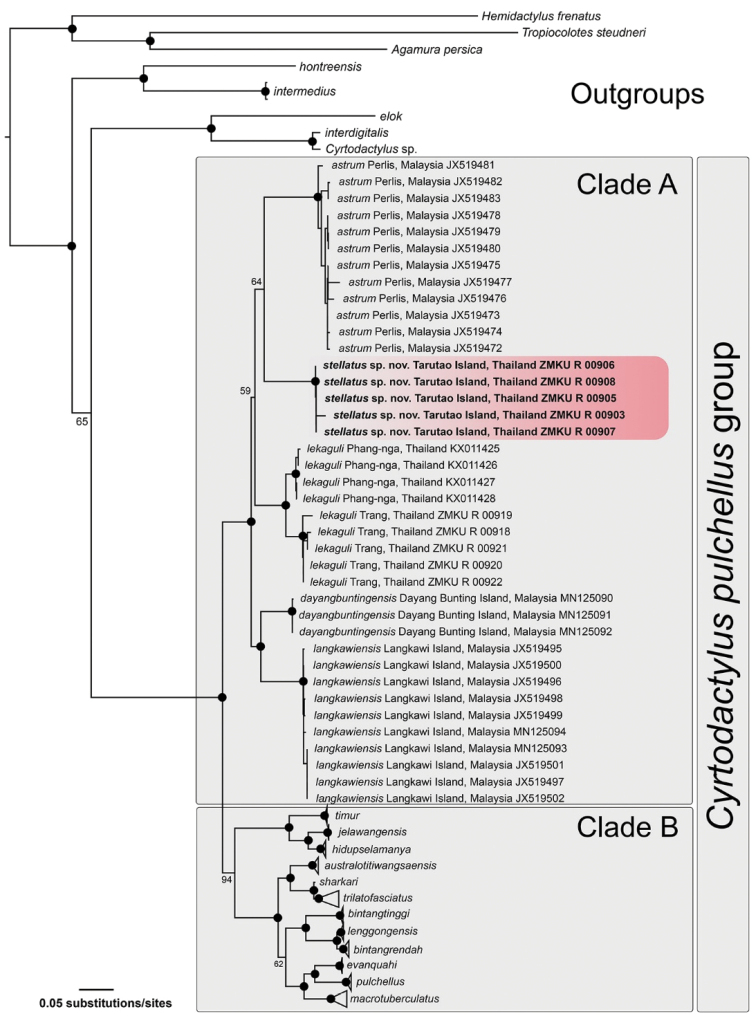
The Maximum Likelihood tree of the *Cyrtodactyluspulchellus* group based on 1,429 bp of the ND2 gene and flanking tRNAs. Support values on branches are ultrafast bootstrap (UFB). Black circles represent nodes strongly supported (UFB ≥ 95).

**Figure 3.**
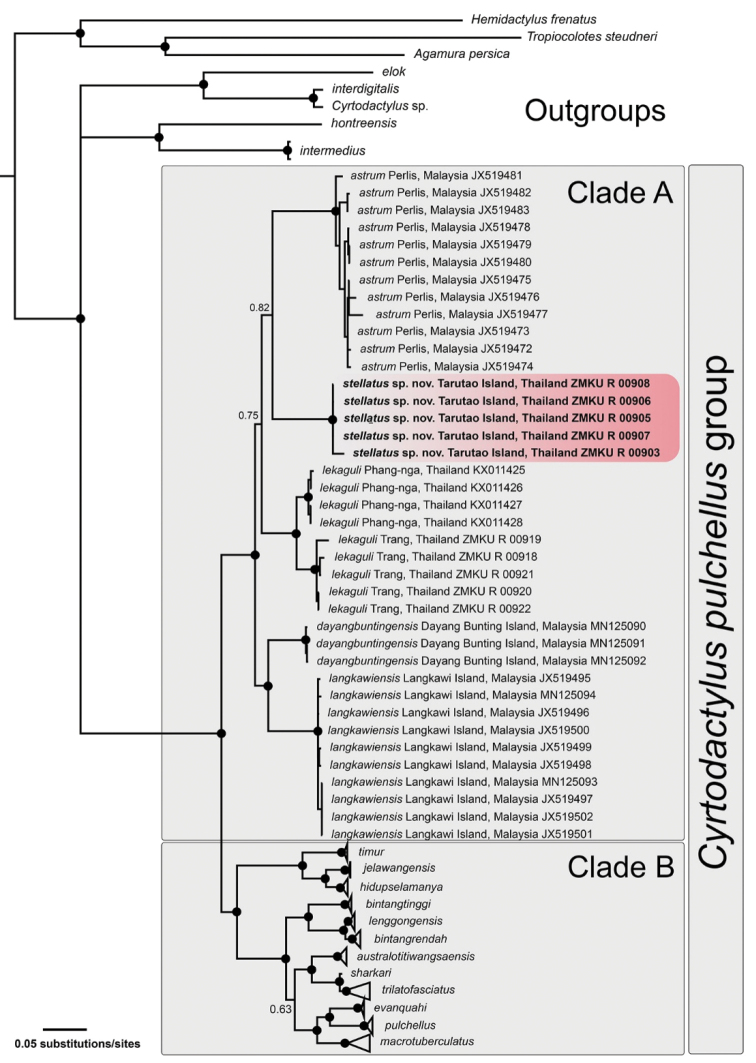
The Bayesian consensus tree of the *Cyrtodactyluspulchellus* group based on 1,429 bp of the ND2 gene and flanking tRNAs. Support values on branches are Bayesian posterior probabilities (BPP). Black circles represent nodes strongly supported (BPP ≥ 0.95).

The topologies of ML and BI analyses were largely concordant. The ML and BI analyses recovered the *C.pulchellus* group as monophyletic with strong support (≥ 95 UFB, ≥ 0.95 BPP) which is comprised of two major clades referred to as Clades A and B (Figs 2, 3). The *Cyrtodactylus* specimens from Tarutao Island represented a strongly supported monophyletic group (≥ 95 UFB, ≥ 0.95 BPP) within Clade A containing *C.astrum*, *C.dayangbuntingensis*, *C.langkawiensis*, and *C.lekaguli*. The Tarutao Island samples were weakly recovered as a sister species to *C.astrum* from the adjacent Peninsular Malaysian mainland (64 UFB, 0.82 BPP). Clade B is composed of all other species including *C.australotitiwangsaensis*Grismer et al., 2012, *C.bintangrendah, C.bintangtinggi*Grismer et al., 2012, *C.evanquahi*Wood et al., 2020, *C.hidupselamanya*Grismer et al., 2016, *C.jelawangensis*Grismer et al., 2014, *C.lenggongensis*Grismer et al., 2016, *C.macrotuberculatus* Grismer and Ahmad, 2008, *C.pulchellus, C.sharkari, C.timur*Grismer et al., 2014 and *C.trilatofasciatus*Grismer et al., 2012. Uncorrected pairwise genetic divergences (*p*-distance) range from 0.00–1.17% within the Tarutao Island specimens and 8.46–12.37% between the Tarutao Island specimens and other species in Clade A (Table 3).

**Table 3.** Percentage uncorrected pairwise genetic divergence (*p*-distances) of *Cyrtodactylusstellatus* sp. nov. and closely related species (Clade A) calculated from 1,429 base pairs of mitochondrial ND2 gene and flanking tRNAs.

	Species	*N*	1	2	3	4	5
1	*Cyrtodactylusstellatus* sp. nov.	5	**0.48 (0.00–1.17)**				
2	*C.astrum*	12	10.50 (9.78–12.37)	**1.37 (0.00–2.97)**			
3	*C.dayangbuntingensis*	3	9.90 (9.56–10.88)	9.86 (9.51–11.21)	**0.14 (0.07–0.22)**		
4	*C.langkawiensis*	10	10.49 (9.86–11.69)	10.19 (9.71–11.59)	7.62 (7.39–7.83)	**0.42 (0.00–0.69)**	
5	*C.lekaguli*	9	9.33 (8.46–10.80)	9.94 (8.98–11.77)	8.58 (8.00–9.59)	9.39 (8.42–10.54)	**2.30 (0.00–4.27)**

### Morphology

**Figure 4.**
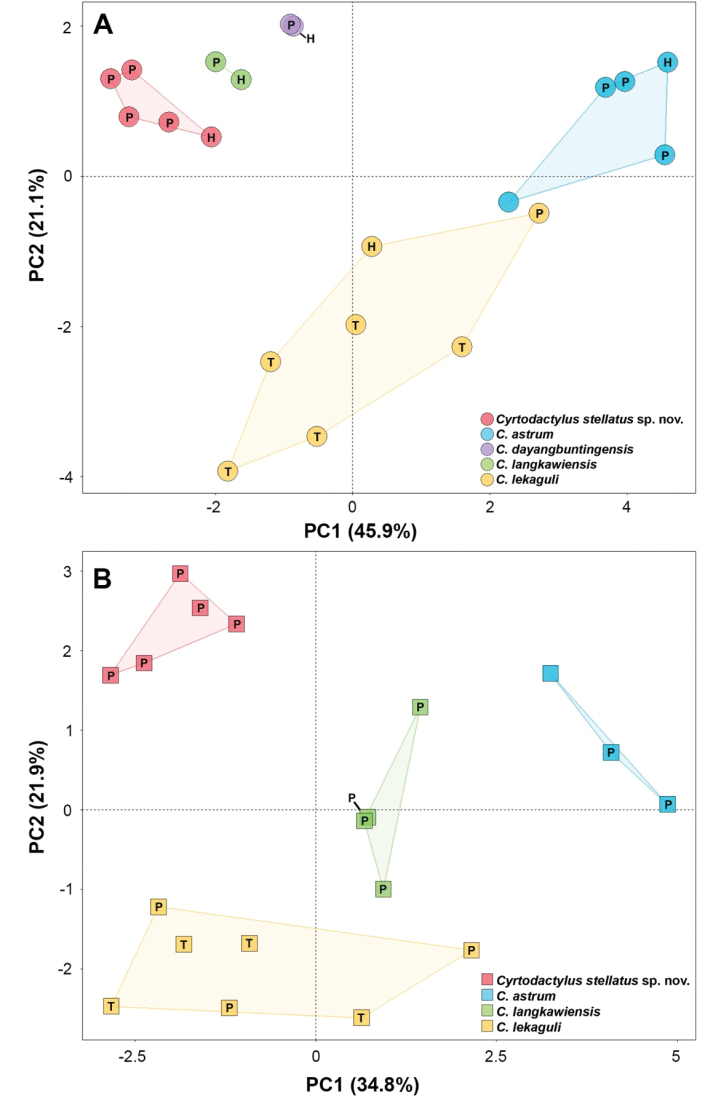
Plots of the first two principal components of *Cyrtodactylusstellatus* sp. nov. and the closely related species in Clade A based on adjusted mensural characters of **A** males and **B** females. The letters in the scatter plots refer to holotype (= H), paratype (= P), and topotype (= T).

The PCA was conducted on members from Clade A. The plots on the first two PC axes showed that the Tarutao Island specimens are clustered separately from other species in both sexes (Fig. 4). In male, the first two principal components explained 66.95% of the morphological variation (Table 4). The first principal component (PC1) accounted for 45.88% of the variation and was heavily loaded on FL_adj_, TBL_adj_, HW_adj_, HD_adj_, EE_adj_, ES_adj_, and EN_adj_; and the PC2 accounted for 21.07% of the variation and was heavily loaded on TW_adj_, AG_adj_, IO_adj_, and EL_adj_. PC analysis of females accounted for 56.74% of the variation in first two components. The PC1 accounted for 34.81% of the variation and was heavily loaded on TBL_adj_, HW_adj_, EE_adj_, ES_adj_, and EN_adj_; and the PC2 accounted for 21.93% of the variation and was heavily loaded on TW_adj_, IO_adj_ and IN_adj_.

**Table 4.** Summary statistics and factor loadings of the first three principal components (PC) of 15 mensural characters for males and females of *Cyrtodactylusstellatus* sp. nov. and its closely related species including *C.astrum*, *C.dayangbuntingensis*, *C.langkawiensis*, and *C.lekaguli*. Bold texts indicate high loadings.

Characters	Males			Females		
	PC1	PC2	PC3	PC1	PC2	PC3
SVL_adj_	0.660	-0.284	0.293	0.374	-0.097	-0.127
TW_adj_	0.436	**0.795**	-0.073	0.380	**0.845**	0.194
FL_adj_	**0.855**	-0.228	-0.051	0.693	0.111	0.003
TBL_adj_	**0.951**	0.098	-0.032	**0.881**	0.181	0.102
AG_adj_	-0.261	-**0.728**	0.364	-0.030	0.157	0.824
HL_adj_	0.459	-0.508	-0.560	0.532	-0.194	0.537
HW_adj_	**0.943**	-0.168	0.177	**0.785**	-0.350	-0.197
HD_adj_	**0.838**	-0.325	0.188	0.526	-0.647	0.170
ED_adj_	0.552	-0.195	-0.491	0.137	-0.086	0.612
EE_adj_	**0.829**	-0.171	0.194	**0.755**	-0.410	0.266
ES_adj_	**0.932**	0.113	0.054	**0.900**	-0.078	-0.271
EN_adj_	**0.875**	0.246	0.142	**0.922**	0.122	-0.263
IO_adj_	0.143	**0.850**	0.435	0.361	**0.854**	0.033
EL_adj_	0.303	**0.770**	-0.417	0.343	0.690	-0.298
IN_adj_	-0.105	0.093	0.560	-0.089	**0.719**	0.238
Eigenvalue	6.882	3.161	1.587	5.222	3.289	1.835
Percentage of variance	45.879	21.073	10.581	34.813	21.928	12.235
Cumulative proportion	45.879	66.952	77.533	34.813	56.741	68.976

The univariate analyses (ANOVA or Kruskal-Wallis test) were significantly different (*p* < 0.05) in most morphological characters among the members of Clade A (except *C.dayangbuntingensis*). In the comparison of adult males, the Tarutao Island population was significantly different from *C.astrum* and *C.lekaguli* in twelve morphological characters (ANOVA or Kruskal-Wallis test, *p* < 0.001–0.006) except AG_adj_, ED_adj_ and IN_adj_ (ANOVA or Kruskal-Wallis test, *p* = 0.051–0.122). Subsequent Tukey’s test or Dunn’s test demonstrated that Tarutao Island population was significantly different from *C.astrum* in SVL_adj_, FL_adj_, TBL_adj_, HL_adj_, HW_adj_, HD_adj_, EE_adj_, ES_adj_, and EN_adj_; and *C.lekaguli* in SVL_adj_, TW_adj_, FL_adj_, HL_adj_, HW_adj_, HD_adj_, EE_adj_, ES_adj_, IO_adj_, and EL_adj_. In adult females, the Tarutao Island population was significantly different from *C.astrum*, *C.langkawiensis* and *C.lekaguli* in nine characters (ANOVA, *p* < 0.001–0.007) except SVL_adj_, TW_adj_, AG_adj_, HL_adj_, ED_adj_ and EL_adj_ (ANOVA or Kruskal-Wallis test, *p* = 0.052–0.631). Subsequent Tukey’s test revealed that the Tarutao Island population was significantly different from *C.astrum* in FL_adj_, TBL_adj_, HW_adj_, HD_adj_, EE_adj_, ES_adj_, and EN_adj_; *C.langkawiensis* in HW_adj_, ES_adj_, EN_adj_, and IN_adj_; and *C.lekaguli* in HD_adj_, IO_adj_, and IN_adj_. Summary pairwise results (Tukey’s test or Dunn’s test) of significant differences in morphological characters for adult males and females of Clade A are shown in Table 5. Additional differences in meristic characters and coloration are discussed in the comparison sections.

**Table 5.** Summary pairwise results of statistically significant characters (Tukey’s test; *p* < 0.05) from 15 mensural characters for males and females of *Cyrtodactylusstellatus* sp. nov. and closely related species (Clade A). Abbreviations are listed in Table 2. Key: * tested by Dunn’s test; M = male; F = female.

		***Cyrtodactylusstellatus* sp. nov.**		***C.astrum***		***C.langkawiensis***	
		M	F	M	F	M	F
*Cyrtodactylusstellatus* sp. nov.	M	–	–	–	–	–	–
	F	–	–	–	–	–	–
*C.astrum*	M	SVL, FL, TBL, HL, HW, HD, EE, ES*, EN	–	–	–	–	–
	F	–	FL, TBL, HW, HD, EE, ES, EN	–	–	–	–
*C.langkawiensis*	M	–	–	–	–	–	–
	F	–	HW, ES, EN, IN	–	HW, IN	–	–
*C.lekaguli*	M	SVL, TW, FL, HL, HW, HD, EE, ES*, IO, EL	–	TW, FL, TBL, HW, EN, IO, EL	–	–	–
	F	–	HD, IO, IN	–	FL, TBL, HW, ES, EN, IO, EL	–	ES

### Taxonomic hypotheses

*Cyrtodactylus* samples from Tarutao Island, Mueang Satun District, Satun Province differed from its congeners in mtDNA, morphometrics and morphological comparisons. These corroborated lines of evidence provide sufficient support to warrant them specific species status and is described as new below.

### Taxonomy

#### Cyrtodactylus stellatus sp. nov.

Taxon classificationAnimaliaSquamataGekkonidae

BE57D219-A831-5896-9B2F-B64B8C41B1A2

http://zoobank.org/F2AF3CB9-F0FE-4749-9785-F57C7CAC021C

**Figure 5.**
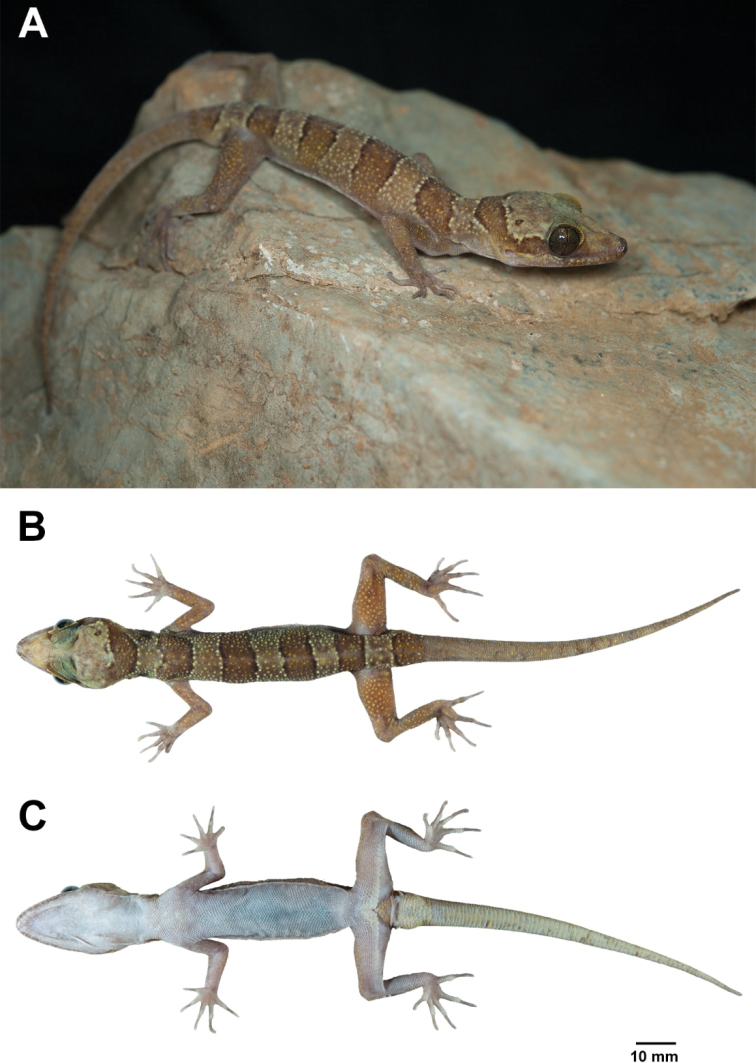
Adult male holotype of *Cyrtodactylusstellatus* sp. nov. (ZMKU R 00905) from Tarutao Island, Satun Province. **A** specimen in life and immediately before preservative: **B** dorsal and **C** ventral views.

**Figure 6.**
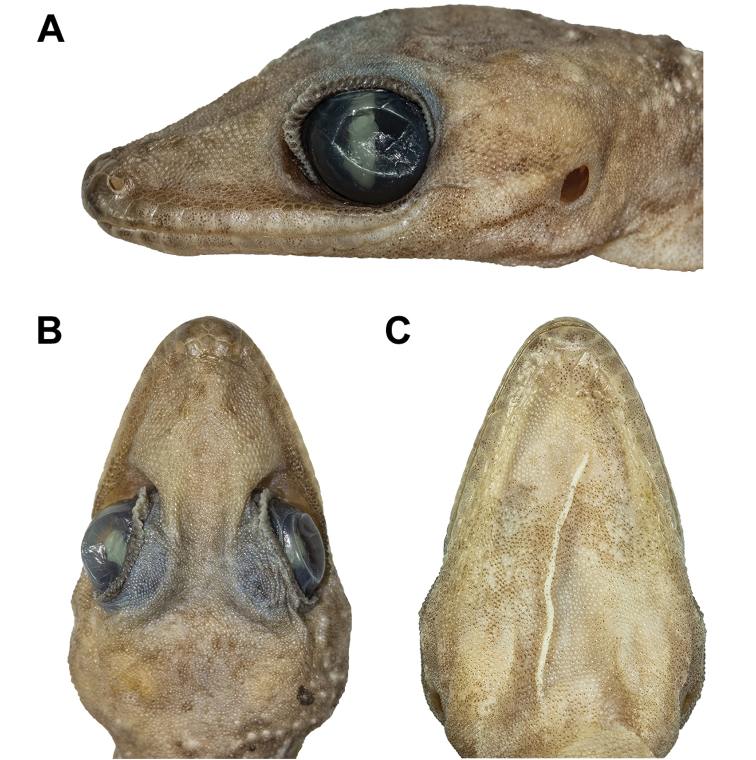
Head of the holotype of *Cyrtodactylusstellatus* sp. nov. (ZMKU R 00905): **A** lateral view of the left side **B** dorsal view, and **C** ventral view.

**Figure 7.**
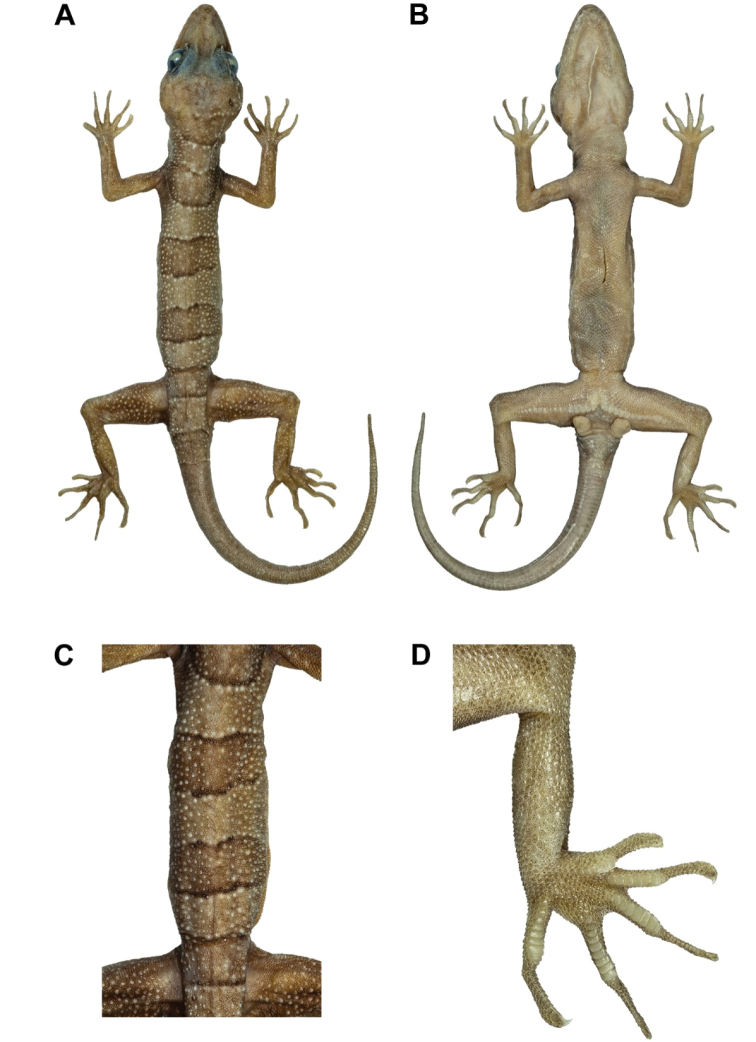
Male holotype of *Cyrtodactylusstellatus* sp. nov. (ZMKU R 00905) in preservation. **A** dorsal and **B** ventral views **C** tuberculation on dorsum, and **D** ventral view of left foot.

**Figure 8.**
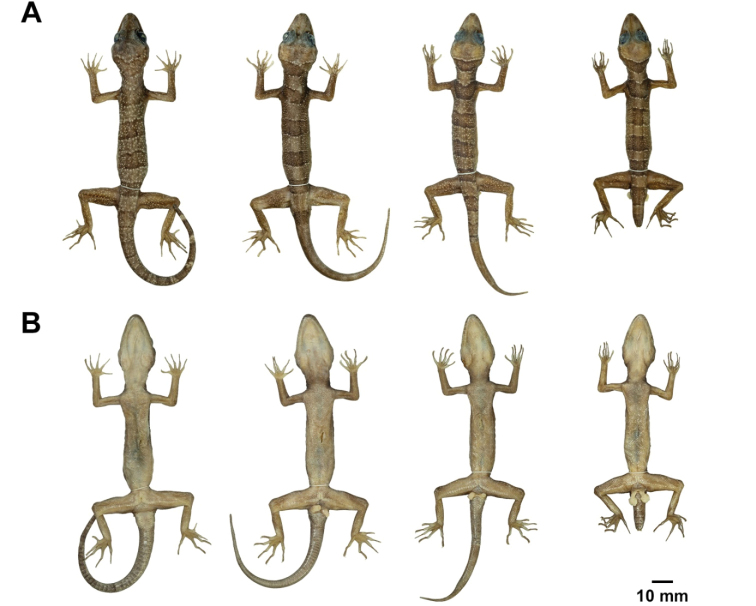
Male paratypes of *Cyrtodactylusstellatus* sp. nov. in preservation. **A** dorsal and **B** ventral views; from left to right: ZMKU R 00903, ZMKU R 00906, ZMKU R 00907, and ZMKU R 00915.

**Figure 9.**
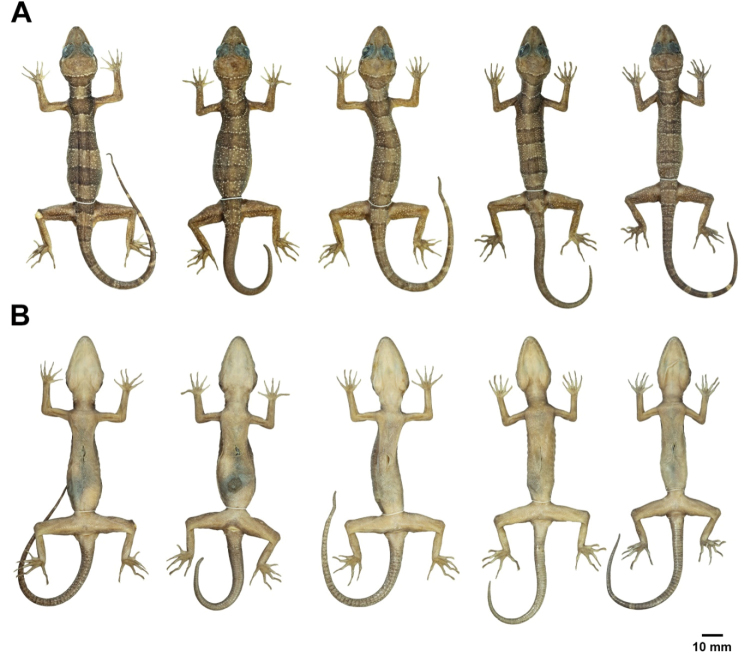
Female paratypes of *Cyrtodactylusstellatus* sp. nov. in preservation. **A** dorsal and **B** ventral views; from left to right: ZMKU R 00899, ZMKU R 00900, ZMKU R 00908, ZMKU R 00909, and ZMKU R 00913.

**Figure 10.**
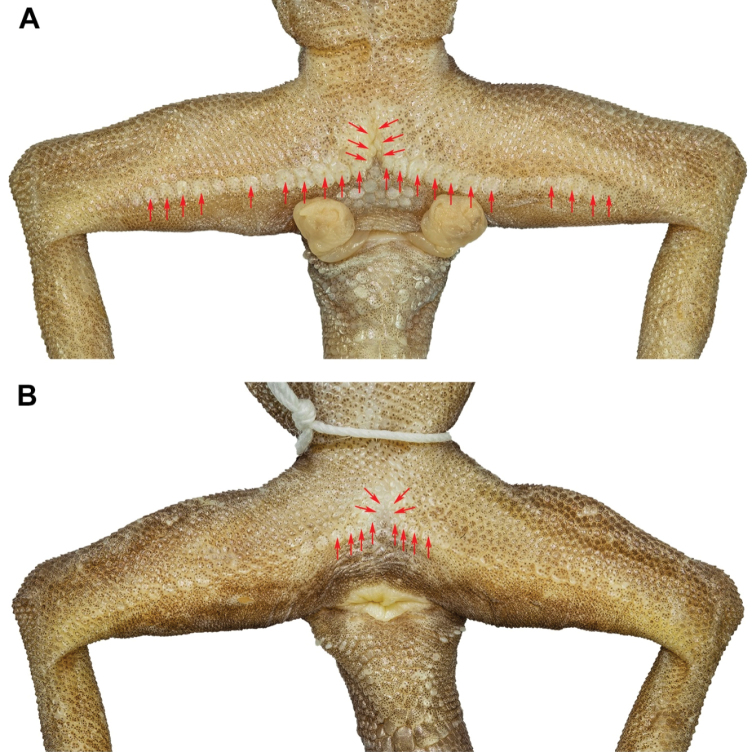
Precloacal region of *Cyrtodactylusstellatus* sp. nov. showing **A** precloacal depression with pore-bearing femoroprecloacal scales in male holotype (ZMKU R 00905), and **B** pore-bearing precloacal scales with lacking depression in female paratype (ZMKU R 00900). Red arrows show pore-bearing scales.

**Figure 11.**
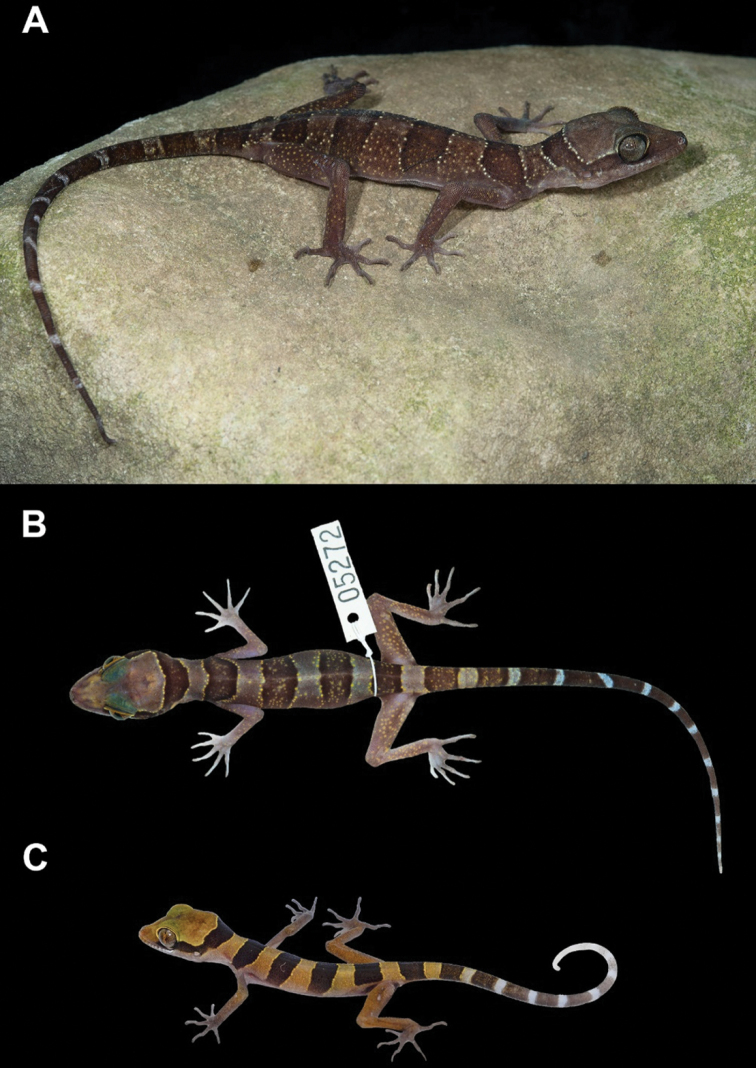
Variation of *Cyrtodactylusstellatus* sp. nov. **A** adult female ZMKU R 00899 having 11 dark caudal bands on the original tail and white caudal bands infused with dark pigmentation **B** immature female ZMKU R 00902 (field number AA 05272) having 12 dark caudal bands on the original tail with immaculate white caudal bands, and **C** juvenile ZMKU R 00917 having light-yellow color on the body and bearing white tail tip.

Figures 5 , 6 , 7 , 8 , 9 , 10 , 11 Stellar Bent-toed Gecko

##### Holotype.

Adult male (ZMKU R 00905, Figs 5–7) collected from Thailand, Satun Province, Mueang Satun District, Tarutao National Park, Tarutao Island, Pha (= cliff) Toe Boo (6°42.185'N; 99°38.895'E; 2 m a.s.l.), on 11 March 2019 by Korkhwan Termprayoon, Anchalee Aowphol, Attapol Rujirawan, Natee Ampai and Siriporn Yodthong.

**Paratypes** (Figs 8–9). Two adult males (ZMKU R 00906–00907) and two adult females (ZMKU R 00908–00909), same data as holotype. One adult female (ZMKU R 00913) same data as holotype except collected on 12 May 2019. One adult male (ZMKU R 00903) and two adult females (ZMKU R 00899–00900), same data as holotype, except collected on 5 November 2017 by Korkhwan Termprayoon, Attapol Rujirawan, Natee Ampai, and Siriporn Yodthong. One adult male (ZMKU R 00915) collected from Thailand, Satun Province, Mueang Satun District, Tarutao National Park, Tarutao Island, Tarutao Outcrop (6°41.617'N; 99°38.796'E; 3 m a.s.l.) on 12 March 2019 by Korkhwan Termprayoon, Anchalee Aowphol, Attapol Rujirawan, Natee Ampai and Siriporn Yodthong.

##### Referred specimens.

ZMKU R 00901 (immature male) and ZMKU R 00902 (immature female) same data as holotype except collected on 5 November 2017 by Korkhwan Termprayoon, Attapol Rujirawan, Natee Ampai, and Siriporn Yodthong. ZMKU R 00904 (immature male) same data as holotype, except collected on 5 April 2018. ZMKU R 00910–00911 (two immature males) and ZMKU R 00912 (immature female) same data as holotype. ZMKU R 00914 (immature female) same data as holotype except collected on 12 May 2019. ZMKU R 00916 (immature male) and ZMKU R 00917 (juvenile) collected from Thailand, Satun Province, Mueang Satun District, Tarutao National Park, Tarutao Island, Tarutao Outcrop (6°41.617'N; 99°38.796'E; 3 m a.s.l.) on 12 March 2019 by Korkhwan Termprayoon, Anchalee Aowphol, Attapol Rujirawan, Natee Ampai and Siriporn Yodthong.

##### Diagnosis.

*Cyrtodactylusstellatus* sp. nov. can be distinguished from all other species of the *C.pulchellus* group by the combination of the following characters: (1) SVL 86.3–95.9 mm in adult males, 86.6–96.1 mm in adult females; (2) 12–15 supralabial and 10–13 infralabial scales; (3) weak tuberculation on body; (4) no tubercles on ventral surfaces of forelimbs, gular region, or in ventrolateral body folds; (5) 32–47 paravertebral tubercles; (6) 19–23 longitudinal rows of dorsal tubercles; (7) 32–40 rows of ventral scales; (8) 20–23 subdigital lamellae on the fourth toe; (9) 24–29 femoroprecloacal pores in adult males; (10) precloacal pores present in adult females; (11) deep precloacal groove in males; (12) dorsum bearing a scattered pattern of white tubercles; (13) four dark dorsal body bands; (14) 10–12 dark caudal bands on original tail; (15) white caudal bands in adults heavily infused with dark pigmentation; and (16) posterior portion of tail in hatchlings and juveniles white.

##### Description of holotype.

Adult male SVL 94.2 mm; head large, moderate in length (HL/SVL 0.29) and wide (HW/HL 0.61), somewhat flattened (HD/HL 0.38), distinct from neck, and triangular in dorsal profile; lores concave anteriorly, inflated posteriorly; frontal and prefrontal regions deeply concave; canthus rostralis rounded anteriorly; snout elongate (ES/HL 0.39), rounded in dorsal profile, laterally constricted; eye large (ED/HL 0.25); ear opening elliptical, moderate in size (EL/HL 0.09), obliquely oriented; eye to ear distance slightly greater than diameter of eye; rostral rectangular, divided dorsally by an inverted Y-shaped furrow, bordered posteriorly by left and right supranasals and internasal, bordered laterally by first supralabials; external nares bordered anteriorly by rostral, dorsally by a large anterior supranasal, posteriorly by two postnasals, ventrally by first supralabial; 13/14 (left/right) rectangular supralabials extending to just beyond upturn of labial margin, tapering abruptly below midpoint of eye; second supralabial slightly larger than first; 11/11 infralabials tapering in size posteriorly; scales of rostrum and lores slightly raised, larger than granular scales on top of head and occiput, those on posterior portion of canthus rostralis slightly larger; scales on occiput intermixed with small tubercles; large, boney frontal ridges bordering orbit confluent with boney, V-shaped, transverse, parietal ridge; dorsal superciliaries elongate, smooth, largest anteriorly; mental triangular, bordered laterally by first infralabials and posteriorly by left and right, trapezoidal postmentals which contact medially for 50% of their length; one row of slightly enlarged, elongate sublabials extending posteriorly to the seventh (left) and fifth (right) infralabials; small, granular, gular scales grading posteriorly into larger, flat, smooth, imbricate, pectoral and ventral scales.

Body relatively short (AG/SVL 0.46) with well-defined, non-tuberculate, ventrolateral folds; dorsal scales small, granular, interspersed with low, regularly arranged, weakly keeled tubercles, smaller intervening tubercles occasionally present; tubercles extend from occiput to caudal constriction, absent from regenerated portion of tail; tubercles on occiput and nape relatively small, those on body largest; approximately 21 longitudinal rows of tubercles at midbody; 36 paravertebral tubercles; 33 flat imbricate ventral scales between ventrolateral body folds; ventral scales larger than dorsal scales; precloacal scales large, smooth; deep precloacal groove.

Forelimbs moderate in stature, relatively short (FL/SVL 0.16); scales on dorsal surfaces of forelimbs granular intermixed with larger tubercles; scales of ventral surface of forearm flat, subimbricate, tubercles absent; palmar scales small, weakly rounded; digits well-developed, inflected at basal, interphalangeal joints; subdigital lamellae rectangular proximal to joint inflection, only slightly expanded distal to inflection; digits narrower distal to joints; claws well-developed, sheathed by a dorsal and ventral scale; the fifth digit broken on left forearm; hind limbs more robust than forelimbs, moderate in length (TBL/SVL 0.19), larger tubercles on dorsal surface of legs separated by smaller juxtaposed scales; ventral scales of thigh flat, smooth, imbricate, larger than dorsal granular scales; ventral, tibial scales flat, smooth, imbricate; a single row of 34 enlarged femoroprecloacal scales extend nearly from knee to knee through precloacal region where they are continuous with enlarged, pore-bearing precloacal scales; 27 separated pore-bearing femoroprecloacal scales (Fig. 10A), forming an inverted T bearing a deep, precloacal groove; six pore-bearing scales bordering groove (three on each side of groove); postfemoral scales immediately posterior to enlarged scale row small, nearly granular, forming an abrupt union with postfemoral scales on posteroventral margin of thigh; plantar scales weakly rounded to flat; digits well developed, inflected at basal, interphalangeal joints; subdigital lamellae proximal to joint inflection rectangular, only slightly expanded distal to inflection; digits narrower distal to joints; claws well-developed, sheathed by a dorsal and ventral scale; 21/22 subdigital lamellae on the 4^th^ toe.

Tail 94.8 mm in length, completely regenerated, 9.2 mm in width at base, tapering to a point; regenerated tail covered with small, smooth, rectangular scales dorsally; base of tail bearing hemipenial swellings; one row of 4/4 medium-sized postcloacal tubercles on each hemipenial swelling; postcloacal scales smooth, flat, large, imbricate.

**Coloration in life (Fig. 5).** Dorsal ground color of head, body, and limbs light-brownish grey; a wide, dark-brown nuchal band bordered anteriorly and posteriorly by thin, creamy-white lines bearing tubercles that extend from the posterior margin of one eye to the posterior margin of other eye; the color of nuchal band and creamy-white lines is faded above left ear opening; four dark-brown body bands between nuchal loop and hind limb insertions that are also bordered anteriorly and posteriorly by thin, creamy-white lines bearing tubercles, first band terminates at shoulders, second and third bands terminate just dorsal of ventrolateral folds, the fourth band terminates at femurs; dark body bands slightly larger than light-colored interspaces; creamy-white to light-yellow tubercles scattered on dorsal surfaces of nape, body, and limbs; one additional dark-brown band posterior to hind limbs; light-brown regenerated tail, bearing yellowish pigment on some scales; ventral surfaces of head smudged with brown; abdomen and limbs beige, with slightly darker, lateral regions.

**Coloration in preservative (Figs 6, 7).** The overall color pattern of head, body, limbs, and tail similar to that in life with some fading. Ground color of head, body, limbs, and dorsum light-brown; dark body bands lighter than in life. Colored tuberculation on dorsum fade to off-white. Tan colored on the ventral surface.

##### Variation.

*Cyrtodactylusstellatus* sp. nov. usually varies in coloration and banding pattern (Figs 8–11; Tables 6, 7). All specimens possess a clear dark-brown nuchal band which is less clearly defined in ZMKU R 00903 and the holotype. In adult females, precloacal pores are present but they lack the precloacal groove (Fig. 10B). Four specimens (ZMKU R 00903, ZMKU R 00907, ZMKU R 00911, and ZMKU R 00913) have prominent light-yellow tubercles scattered on the dorsum and limbs. Male paratype (ZMKU R 00907) has continuous pore-bearing femoroprecloacal scales. Original tails (ZMKU R 00899, ZMKU R 00901–00902, ZMKU R 00910, ZMKU R 00912, and ZMKU R 00916) have 10–12 dark caudal bands (Fig. 11A, B), light bands diffused with dark pigment in adults (immaculate in immature and juvenile), subcaudal scales transversely enlarged and shallow caudal furrows. Male paratypes have a single row of 3–4L/2–4R postcloacal tubercles on each hemipenial swelling except ZMKU R 00907 which has two rows of 4/5 on each side. This character in female paratypes is very small, and a single row of 2–4/2–4 on each side at the base of tail.

**Table 6.** Descriptive measurement (millimeters), meristic (left/right) and color pattern characters of the type series of *Cyrtodactylusstellatus* sp. nov. Key: H = holotype; P = paratype; M = male; F = female; / = data unavailable or unapplicable; b = broken; r = regenerated. Morphological abbreviations are defined in Table 2.

	ZMKU R 00905	ZMKU R 00903	ZMKU R 00906	ZMKU R 00907	ZMKU R 00915	ZMKU R 00899	ZMKU R 00900	ZMKU R 00908	ZMKU R 00909	ZMKU R 00913
	H	P	P	P	P	P	P	P	P	P
Sex	M	M	M	M	M	F	F	F	F	F
SVL	94.2	95.9	94.6	87.9	86.3	96.1	93.6	94.8	90.4	86.6
TL	94.8r	96.8r	92.3r	69.0r	18.5b	124.4	70.5r	107.5r	81.7r	102.3r
TW	9.2	9.7	9.2	8.4	7.5	7.6	9.1	9.1	10.3	7.1
FL	15.4	15.3	15.1	14.4	14.1	14.9	14.5	15.9	15.6	14.8
TBL	18.1	18.3	17.8	17.4	17.6	17.7	17.3	18.1	17.8	16.5
AG	43.3	43.7	43.9	44.4	39.6	46.0	44.5	44.5	45.4	39.9
HL	27.6	26.3	27.0	25.2	24.8	26.5	26.9	27.3	26.4	25.7
HW	16.7	16.3	17.3	15.4	14.7	16.4	16.8	17.2	15.7	15.2
HD	10.4	9.6	10.8	9.2	8.7	10.4	10.2	10.1	9.4	9.2
ED	6.8	6.7	6.7	5.9	5.8	6.4	6.8	6.6	6.6	5.6
EE	7.1	7.6	7.2	6.3	6.2	6.9	7.0	7.0	6.7	6.8
ES	10.8	10.7	10.9	9.8	9.8	10.9	10.8	10.7	10.4	10.1
EN	8.5	8.3	8.3	7.8	7.5	8.2	8.3	8.5	8.1	7.9
IO	5.9	6.2	6.6	5.8	5.5	6.0	6.4	6.1	5.7	6.2
EL	2.4	2.5	2.2	2.0	2.0	2.0	2.8	2.7	2.1	2.3
IN	2.9	3.2	3.5	3.1	3.0	3.8	3.3	3.2	3.2	2.6
SL	13/14	14/13	15/12	13/14	13/12	13/13	13/13	13/12	12/12	13/13
IL	11/11	10/10	11/12	12/12	11/11	11/11	12/11	12/12	10/11	11/11
PVT	36	35	41	38	43	38	40	40	40	47
LRT	21	20	21	19	22	20	21	19	22	22
VS	33	37	36	35	36	37	39	37	37	36
4TL	21/22	21/21	21/21	21/20	23/22	22/22	21/21	22/23	22/20	20/20
FPP in adult males	27	25	24	29	27	/	/	/	/	/
PP in adult females	/	/	/	/	/	15	12	14	11	11
BB	4	4	4	4	4	4	4	4	4	4
DCB	/	/	/	/	/	11	/	/	/	/
Body band/ interspace ratio	1.12	1.20	1.04	1.10	1.68	1.07	1.03	1.23	1.06	0.92
Precloacal groove	Deep	Deep	Deep	Deep	Deep	Absent	Absent	Absent	Absent	Absent
Femoroprecloacal pores continuous	No	No	No	Yes	No	/	/	/	/	/
Tuberculation	Weak	Weak	Weak	Weak	Weak	Weak	Weak	Weak	Weak	Weak
Tubercles on ventral surface of forelimb	No	No	No	No	No	No	No	No	No	No
Tubercles in gular region	No	No	No	No	No	No	No	No	No	No
Ventrolateral fold tuberculate	No	No	No	No	No	No	No	No	No	No
Dorsum bearing scattered pattern of white tubercles	Yes	Yes	Yes	Yes	Yes	Yes	Yes	Yes	Yes	Yes
Hatchlings/ juveniles with white tail tip	/	/	/	/	/	/	/	/	/	/
Adult posterior caudal region white	/	No	/	/	/	No	/	No	/	/
White caudal bands in adults immaculate	/	No	/	/	/	No	/	No	/	No

**Table 7.** Descriptive meristic (left/right) and color pattern characters of the referred specimens of *Cyrtodactylusstellatus* sp. nov. Key: RF = referred specimen; IM-M = immature male; IM-F = immature female; J = juvenile; / = data unavailable or unapplicable. Morphological abbreviations are defined in Table 2.

	ZMKU R 00901	ZMKU R 00902	ZMKU R 00904	ZMKU R 00910	ZMKU R 00911	ZMKU R 00912	ZMKU R 00914	ZMKU R 00916	ZMKU R 00917
	RF	RF	RF	RF	RF	RF	RF	RF	RF
Age	IM-M	IM-F	IM-M	IM-M	IM-M	IM-F	IM-F	IM-M	J
SVL	77.4	68.4	72.5	82.5	81.8	73.8	81.9	79.9	43.1
SL	12/12	13/13	12/12	12/13	14/14	14/14	13/13	13/14	/
IL	10/11	11/12	10/11	11/11	10/12	13/11	10/11	10/11	/
PVT	38	38	32	41	41	41	42	40	/
LRT	20	21	19	22	19	19	22	23	/
VS	34	40	34	37	32	37	38	37	/
4TL	22/21	22/22	21/20	21/21	20/21	23/23	22/21	21/22	/
FFP in adult males	/	/	/	/	/	/	/	/	/
PP in adult females	/	/	/	/	/	/	/	/	/
BB	4	4	4	4	4	4	4	4	4
DCB	11	12	/	11	/	10	/	11	/
Body band/ interspace ratio	1.35	1.36	1.40	1.09	0.99	1.44	1.22	1.39	/
Precloacal groove	/	/	/	/	/	/	/	/	/
Tuberculation	Weak	Weak	Weak	Weak	Weak	Weak	Weak	Weak	Weak
Tubercles on ventral surface of forelimb	No	No	No	No	No	No	No	No	No
Tubercles in gular region	No	No	No	No	No	No	No	No	No
Ventrolateral fold tuberculate	No	No	No	No	No	No	No	No	No
Dorsum bearing scattered pattern of white tubercles	Yes	Yes	Yes	Yes	Yes	Yes	Yes	Yes	No
Hatchlings/juveniles with white tail tip	No	No	No	No	No	No	No	No	Yes
Adult posterior caudal region white	/	/	/	/	/	/	/	/	/
White caudal bands in adults immaculate	/	/	/	/	/	/	/	/	/

In life, the juvenile (ZMKU R 00917; SVL 43.1 mm) had a body pattern similar to the adults but with less prominent tuberculation, brownish yellow ground color of body, dark body bands are bordered by yellow lines, some bearing tubercles, the original tail has approximately 10 dark caudal bands, the posterior portion of tail is white, and light caudal bands are immaculate (Fig. 11C).

##### Distribution.

**Figure 12.**
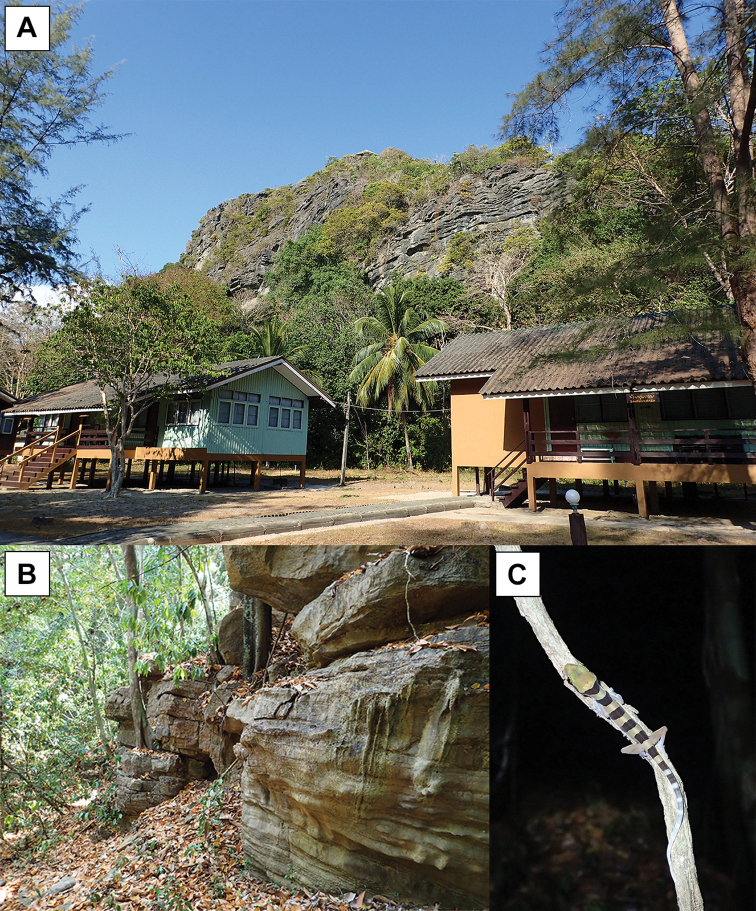
Habitat of *Cyrtodactylusstellatus* sp. nov. at the type locality, Tarutao Island, Satun Province, Thailand. **A** Pha Toe Boo karst formation **B** karst microhabitat structure and **C** vegetation (vine) used by a juvenile in karst habitat.

*Cyrtodactylusstellatus* sp. nov. is currently known only from Tarutao Island, Satun Province, Thailand (Figs 1, 12A).

##### Natural history.

All specimens of *C.stellatus* sp. nov. were collected from a karst forest at night (1950–2100 h) with temperatures between 27.1–32.2 °C and relative humidity between 71.4–93.0%. The specimens were found on karst walls, within karst crevices and on nearby karst boulders. Some specimens occurred on tree trunks or vines near the karst formations (Fig. 12). The holotype was found on a karst wall approximately 1 m above the ground within karst forest. Eight specimens (ZMKU R 00900, ZMKU R 00906, ZMKU R 00908, ZMKU R 00911–00912, ZMKU R 00913, and ZMKU R 00915–00916) were found on karst walls from 0.5–3.0 m above the ground. ZMKU R 00907, ZMKU R 00910, and ZMKU R 00914 were found in karst crevices. Three specimens (ZMKU R 00901, ZMKU R 00903, and ZMKU R 00909) were found on karst boulders. Four specimens (ZMKU R 00899, ZMKU R 00902, ZMKU R 00904, and ZMKU R 00917) were perched on vegetation near karst walls or karst boulders.

Two gravid females (ZMKU R 00899–00900) were collected in November 2017 and contained two eggs (externally visible). The juvenile was found on a vine in May 2019. *Cyrtodactylusstellatus* sp. nov. appears to be nocturnal and sympatric with two other gekkonids, *Gehyramutilata* Wiegmann, 1834 and the diurnal species *Cnemaspistarutaoensis*Ampai et al., 2019.

##### Etymology.

The specific epithet *stellatus* is Latin word, meaning starry or starred, and refers to scattered pattern of light-colored tubercles on dorsum and limbs. The name corresponds with the sister taxon *C.astrum* that shared similar diagnostic character (scattered light-colored tubercles pattern on dorsum).

##### Comparison.

*Cyrtodactylusstellatus* sp. nov. can be distinguished from other species in the *C.pulchellus* group by having a combination of weak tuberculation on the body; no tubercles on ventral surface of forelimbs, gular region, or in ventrolateral body folds; 19–23 longitudinal tubercle rows; 32–40 ventral scales; 20–23 subdigital lamellae on the fourth toe; 24–29 femorprecloacal pores in males; deep precloacal groove in males; 11–15 precloacal pores in females; scattered pattern of white, cream or light-yellow tubercles on dorsum; 10–12 dark caudal bands on original tail; white caudal bands on original tail infused with dark pigmentation in adults; and juveniles with white tail tip. Additional comparisons between *C.stellatus* sp. nov. and other species in the *C.pulchellus* group are in Table 8.

**Table 8.** Diagnostic characters of *Cyrtodactylusstellatus* sp. nov. and its related species within the *C.pulchellus* group. W = weak; P = prominent; / = data unavailable. Some information was collected from the following literature (Grismer et al. 2012, 2014, 2016; Quah et al. 2019, Wood et al. 2020, and Termprayoon et al. 2021).

	Clade A					Clade B											
	*stellatus* sp. nov.	*astrum*	*dayangbuntingensis*	*langkawiensis*	*lekaguli*	*australotitiwangsaensis*	*bintangrendah*	*bintangtinggi*	*evanquahi*	*hidupselamanya*	*jelawangensis*	*lenggongensis*	*macrotuberculatus*	*pulchellus*	*sharkari*	*timur*	*trilatofasciatus*
Sample size	10	13	3	10	16	12	6	14	3	14	4	4	39	13	1	5	6
Maximum SVL	96.1	108.3	99.0	99.8	108.3	120.1	114.4	111.1	96.0	102.7	119.8	103.1	117.9	114.1	100.1	120.5	122.2
SL	12–15	10–12	12–14	9–12	10–12	9–12	8–12	9–13	9 or 10	9–11	10–12	10 or 11	9–12	11	10–12	9–12	9–13
IL	10–13	9–12	10–11	8–10	9–11	9–13	8 or 10	8–11	9 or 10	8–10	8–10	8–10	9–11	10	8–11	8–11	7–11
PVT	32–47	40–57	35–36	34–44	30–50	37–45	36–44	31–42	31–34	33–43	38–43	36–41	36–42	31	34–38	39–48	34–49
LRT	19–23	20–29	20–22	21–25	20–25	22–30	22–25	21–26	18–23	19–24	23–25	22–25	19–27	22–26	24	21–24	23–27
VS	32–40	31–46	36–39	38–43	30–43	32–40	31–39	36–40	29–33	26–33	31–36	32 or 33	17–28	29–34	41	31–40	33–36
4TL	20–23	20–24	21–23	19–21	20–25	21–25	21–24	21–24	22–23	19–24	21–24	20–23	19–23	21–26	24	21–25	22–27
FPP in adult males	24–29	28–38	26–29	30	30–40	39–45	41–46	37–41	32–36	17–22	36	39–41	28–42	33–39	46	21 or 22	41–46
PP in adult females	11–15	Absent	Absent	Absent	Absent	Absent	Absent	Absent	Absent	Absent	Absent	Absent	Absent	Absent	Absent	Absent	Absent
No. of body bands	4	4	4	4 or 5	4 or 5	3(1) or 4	4	3(1) or 4	6 or 7	4	4	4 or 5	3–4	4	4	4	3
Body band/ interspace ratio	0.92–1.68	0.98–2.07	0.75	0.75–1.00	0.86–2.00	1.00–2.00	1.00–1.25	1.00–1.25	0.82–1.10	1.00–1.25	1.00–1.50	0.50–1.25	0.95–1.74	0.75–1.25	1.75	1.00–1.25	2.00–2.75
DCB	10–12	13 or 14	>7	11–16	12–14	7–8	8 or 9	8–10	9–11	8–10	10	14	7–10	8–10	7	8–10	6–7
Precloacal groove in males	Deep	Deep	Deep	Deep	Deep	Deep	Deep	Deep	Shallow	Deep	Deep	Deep	Deep	Deep	Shallow	Deep	Deep
Femoroprecloacal pores continuous	Both	Yes	Yes	Yes	Yes	Yes	Yes	Yes	Yes	Yes	Yes	Yes	Yes	Yes	Yes	Yes	Yes
Tuberculation	W	W	W	W	W	P	P	P	P	W	P	W	P	P	W	W	P
Tubercles on ventral surface of forelimb	No	No	No	No	No	No	No	No	No	No	Yes	No	Yes	No	No	No	No
Tubercles in gular region	No	No	No	No	No	No	No	No	No	No	No	No	Yes	No	No	No	No
Ventrolateral fold tuberculate	No	No	No	No	No	No	Yes	No	No	No	No	No	Yes	No	No	No	No
Dorsum bearing scattered pattern of white tubercles	Yes	Yes	Yes	No	No	No	No	No	No	No	No	No	No	No	No	No	No
Hatchlings/juveniles with white tail tip	Yes	Yes	Yes	Yes	Yes	No	No	No	Yes	Yes	Yes	/	No	No	/	No	No
Adult posterior caudal region white	No	No	No	No	No	No	No	No	Yes	Yes	No	No	No	No	No	No	No
White caudal bands in adults immaculate	No	No	No	No	No	Yes	Yes	Yes	No	Yes	No	Yes	No	Yes	Yes	Yes	Yes

Based on phylogenetic tree, *C.stellatus* sp. nov. is embedded in Clade A along with *C.astrum, C.dayangbuntingensis, C.langkawiensis*, and *C.lekaguli*. It can be distinguished from all four species by having smaller maximum SVL of 96.1 mm (vs. 108.3 mm in *C.astrum*, 99.0 mm in *C.dayangbuntingensis*, 99.8 mm in *C.langkawiensis*, and 108.3 in *C.lekaguli*); 24–29 femoroprecloacal pores in males (vs. 28–38 in *C.astrum*, 30 in *C.langkawiensis*, and 30–40 in *C.lekaguli*); 11–15 precloacal pores in females (vs. absent in *C.astrum, C.dayangbuntingensis, C.langkawiensis*, and *C.lekaguli*); scattered pattern of white, cream or light-yellow tubercles on dorsum (vs. absent in *C.langkawiensis*, and *C.lekaguli*); the ratio of dark body bands to the light color interspaces 0.92–1.68 (vs. 0.75 in *C.dayangbuntingensis*); 10–12 dark caudal bands (vs. 13 or 14 in *C.astrum*).

## Discussion

The discovery of *C.stellatus* sp. nov. brings the total number of species in the *C.pulchellus* group to 17, of which four have been reported from Thailand. This new species is only known from karst habitats on Tarutao Island and seems to have a narrow geographic distribution (endemic to Tarutao Island). Molecular analyses recovered it as the sister taxon to *C.astrum* and is closely related to *C.dayangbuntingensis, C.langkawiensis*, and *C.lekaguli*. Although *C.stellatus* sp. nov. showed a similar morphological pattern to its sister species, morphological analyses and comparisons of meristic characters revealed that this new species is clearly different from its congeners species of *Cyrtodactylus*. Among *Cyrtodactylus*, most useful diagnostic characters are associated with the femoral and precloacal pores (Harvey et al. 2015). These characters are easily detected in males, but those in females are superficial and only found in some species (e.g., *C.marmoratus* Gray, 1831; *C.psarops*Harvey et al., 2015; *C.sworderi* Smith, 1925). We found differences in pore-bearing scales between *C.stellatus* sp. nov. and other species in the *C.pulchellus* group, that proved to be useful in distinguishing among species. Members of the *C.pulchellus* group mostly possess a continuous series of enlarged, pore-bearing femoroprecloacal scales in males, but *C.stellatus* sp. nov. presents a discontinuous row of femoroprecloacal pores except one individual (ZMKU R 00907), which has a continuous series. Moreover, the presence of precloacal pores were found in females of *C.stellatus* sp. nov., which has not been reported in the other species (Grismer et al. 2012; Quah et al. 2019; Wood et al. 2020, Termprayoon et al. 2021).

In addition, we found that the reported sampling localities of *C.lekaguli* (ZMKU R 00720–00723) were incorrectly stated as “Thailand, Changwat Province, Takua Pa District, Phangnga” in previous studies (i.e., Grismer et al. 2016; Quah et al. 2019; Wood et al. 2020, Termprayoon et al. 2021). Therefore, we corrected the sampling localities to “Thailand, Phang-nga Province, Mueang Phang-nga District” (see Table 1).

The discovery of this new species suggests that undiscovered species of the *C.pulchellus* group may still occur in southern Thailand where there are still numerous unexplored karst areas. Additional surveys are needed to determine the extent of the geographic range of *C.stellatus* sp. nov. and the *C.pulchellus* group in as a whole in the region.

## Supplementary Material

XML Treatment for Cyrtodactylus stellatus

## Appendix I

**Specimens examined**

*Cyrtodactylusastrum*: **Peninsular Malaysia**, Perlis, Gua Wang Burma: LSUHC 09928 (female) and LSUHC 100075 (male).

*Cyrtodactyluslekaguli*: **Thailand**, Trang Province, Na Yong District: ZMKU R 00918, THNHM 017781, THNHM 017784, THNHM 017787, THNHM 017791 (5 males), and ZMKU R 00919, ZMKU R 00921, THNHM 017694, THNHM 017777, THNHM 017788, THNHM 017790 (6 females).
